# Recent advances in the management of gastric adenocarcinoma patients

**DOI:** 10.12703/r/12-2

**Published:** 2023-02-21

**Authors:** Jane E Rogers, Jaffer A Ajani

**Affiliations:** 1 Pharmacy Clinical Programs, U.T. M.D. Anderson Cancer Center, Houston, TX, USA; 2 Department of Gastrointestinal Medical Oncology, U.T. M.D. Anderson Cancer Center, Houston, TX, USA

**Keywords:** gastric neoplasms, programmed death-1, trastuzumab deruxtecan, fibroblast growth factor receptor, claudin

## Abstract

Gastric adenocarcinomas are a significant cause of cancer and cancer death, globally. The curative approach for those with diagnosed localized disease is with surgical resection and an adjunctive approach of perioperative chemotherapy, postoperative adjuvant therapy, or postoperative chemoradiation. Unfortunately, a universal standard approach is lacking for adjunctive therapy which in part has limited the progress achieved in this area. Metastatic disease is common in the Western world at diagnosis. Metastatic disease is treated palliatively with systemic therapy. Targeted therapy has stalled in approvals in gastric adenocarcinomas. Recently, we have seen the exploration of promising targets along with the addition of immune checkpoint inhibitors in select patients. Here, we review recent advances seen in gastric adenocarcinomas.

## Introduction

Gastric adenocarcinoma (GAC) continues to be a leading cancer and major cause of cancer deaths with high variability in prevalence dependent on geographic regions^1^. There are familial risks (e.g., hereditary non-polyposis colorectal cancer and Li–Fraumeni syndrome), associations with certain diets (e.g., high salt intake), and environmental factors (e.g., *Helicobacter pylori* infection and smoking). GACs are classified anatomically (cardia or non-cardia) and histologically (disuse or intestinal) and are starting to be differentiated on various molecular pathways (e.g., Epstein–Barr virus). Biomarker evaluation has been limited until recently. All patients should be reviewed to determine the presence of microsatellite instability-high/deficient mismatch repair. For those with advanced, unresectable disease, human epidermal growth factor receptor 2 (HER-2) and programmed death-ligand 1 (PD-L1) expression level via a combined positive score (CPS) should be evaluated to determine treatment options. Unfortunately, outcomes remain poor with standard approaches. Recent investigation shows promising targets. Here, we review the current treatment and future steps in GAC.

## Main discussion

In patients with localized GAC, cure cannot be achieved without surgical resection which includes primary tumor resection with negative margins along with adequate lymphadenectomy. Exceptions are successfully endoscopically treated GACs in the very early stage. In addition to surgical resection, localized disease is further treated with perioperative chemotherapy, postoperative adjuvant chemotherapy, or postoperative chemoradiation. The decision on which an adjunctive treatment approach should be considered is based entirely on the preferred geographic practices. Unfortunately, a worldwide standard approach has yet to be established and this confounds and limits the progress in this area. These approaches were established via the Intergroup 116, MAGIC, ACTS-GC, FLOT-AIO, and CLASSIC trials among others^1–6^. Many of these studies highlight an important issue that many patients are not able to complete postoperative therapy. For example, fewer than 50% of patients completed the postoperative allotted cycles in the MAGIC and FLOT-AIO trials^3,5^. Currently, we recommend that localized GAC be treated at a high-volume center and be reviewed in a multidisciplinary conference. We believe that an attempt to complete all systemic therapy prior to surgery would be more rewarding. Clinical trial enrollment must remain a priority in this setting as outcomes remain poor. Additionally, we must continue to obtain more insight molecularly to characterize the heterogenous nature of GACs.

In recent years, a unique group of patients with localized GAC has been identified with MSI-H/dMMR (microsatellite instability-high/mismatch repair-deficient) which can occur in about 15% of localized GACs^7,8^. Patients with MSI-H/dMMR GAC have had dramatic results with immune checkpoint therapy, as evident with MSI-H/dMMR metastatic colorectal cancer^9–12^. Programmed death-1 blockade is under investigation in localized MSI-H/dMMR solid tumors^13,14^. Lumish *et al.* recently reported the results of 12 localized stage II or III MSI-H/dMMR rectal cancer patients who achieved 100% objective response rate (ORR) with dostarlimab, an anti-PD-1 agent^13^. This phase 2 trial reported on seven patients who completed induction therapy. All seven achieved a complete clinical response and are undergoing observation without chemoradiation and surgery. Similarly, Ludford *et al.* reported results of a phase 2 evaluation of pembrolizumab, an anti-PD-1 agent, in patients with MSI-H/dMMR localized solid tumor^14^. In 30 patients evaluated, the ORR was 77%. Six patients underwent surgery, 50% had a pathological complete response (pCR). A non-operative approach (pembrolizumab for 12 months) was chosen in 15 patients. One-year organ sparing was noted in both (n = 2) evaluable patients at the time of the report. Encouragingly, André *et al.* recently provided results of the GERCOR NEONIPIGA phase 2 trial, which evaluated nivolumab in combination with ipilimumab, an anti-cytotoxic T-lymphocyte-associated protein 4 agent, in patients with localized MSI-H/dMMR gastroesophageal adenocarcinoma^15^. These patients (n = 32) were given neoadjuvant nivolumab every 2 weeks (× 6 doses) and ipilimumab every 6 weeks (× 2 doses) and then taken to surgery. pCR was reported at 59% (17/29 resected with R0 resection), and another 14% had less than 10% residual tumor per tumor bed. The NICHE-2 study on patients with stage II/III MSI-H/dMMR colorectal cancer (n = 112) showed similar pCR of 67% when these patients were given nivolumab + ipilimumab neoadjuvantly prior to surgery^16^. Given the small numbers treated in these studies, we feel that a basket trial agnostic for tumor type but with MSI-H/dMMR is likely the best option to make progress more quickly but these early evaluations clearly show a different path needed for those with MSI-H/dMMR disease. The morbidity of chemoradiation and surgery for GAC remains a concern. If avoidance of these measures is possible in some patients, we encourage continued study and enrollment in a clinical trial for those found to have localized MSI-H/dMMR GAC.

In Western countries, GACs are often diagnosed in the advanced setting as adequate screening guidelines are not in place for these areas^1^. Treatment advancements in the metastatic setting have been minor until recently. The traditional approach for years in the metastatic setting is to recommend a combination chemotherapy regimen of fluoropyrimidine plus platinum (oxaliplatin or cisplatin) for front-line therapy. Trastuzumab, an anti-HER-2 agent, was the first (and, for many years, the only) targeted agent approved for metastatic GAC. Trastuzumab was approved based on results of the ToGA trial, which showed improvement for those with HER-2-positive disease (~20%) when trastuzumab was added to fluoropyrimidine + platinum therapy^17^. The RAINBOW^18^ and REGARD^19^ trials published in 2014 led to the approval of ramucirumab, an anti-vascular endothelial growth factor receptor (VEGF) agent, plus paclitaxel and ramucirumab monotherapy in the second-line advanced GAC setting, respectively. Ramucirumab remains a marginal agent for GAC. Outside of these two agents, targeted therapy failed often in GAC studies. Recently, immune checkpoint therapies and an additional anti-HER-2 targeted agent have been approved.

Immune checkpoint therapy in the front-line setting has become relevant in advanced GAC through phase 3 trials of nivolumab, pembrolizumab, and sintilimab^20–25^. These trials unfortunately showed varying results. Thus, we still need clarity surrounding upfront immune checkpoint therapy.

Amongst these phase 3 trials was CHECKMATE 649, a global phase 3 trial. CHECKMATE 649 evaluated nivolumab with fluoropyrimidine plus oxaliplatin, nivolumab plus ipilimumab, or fluoropyrimidine plus oxaliplatin in the front-line HER-2-negative advanced GAC setting^20^. Those with CPS of at least 5 showed improvement in median overall survival (OS) and median progression-free survival (PFS) with the nivolumab combination (median OS of 14.4 months with nivolumab plus chemotherapy vs. 11.1 months of chemotherapy alone, *P* <0.0001; median PFS of 7.7 months nivolumab plus chemotherapy vs. 6.1 months of chemotherapy alone, *P* = 0.0073). Those with CPS of less than 5 did not show improvement. KEYNOTE-062, a global phase 3 trial, evaluated pembrolizumab, pembrolizumab plus fluoropyrimidine plus cisplatin, or chemotherapy alone in patients with HER-2-negative advanced GAC. Patients had to be PD-L1-positive (PD-L1 ≥1)^21^. In those with CPS of at least 1, pembrolizumab monotherapy was not superior to chemotherapy. Pembrolizumab plus chemotherapy was not superior to chemotherapy alone for OS or PFS in patients with CPS of 1 or greater (median OS of 12.5 vs. 11.1 months; *P* = 0.05; median PFS of 12.5 vs. 11.1 months; *P* = 0.05). Pembrolizumab monotherapy prolonged OS compared with chemotherapy in patients with CPS of at least 10 (median OS of 17.4 vs. 10.8 months). Pembrolizumab plus chemotherapy was not superior in OS or PFS for those with CPS of at least 10 compared with chemotherapy alone (median OS of 12.3 vs. 10.8 months; *P* = 0.16). KEYNOTE-811, a global phase 3 trial, evaluated the addition of pembrolizumab or placebo to trastuzumab + fluoropyrimidine + cisplatin or oxaliplatin in HER-2-positive patients^22^. Initial findings show benefit to the addition of pembrolizumab. Pembrolizumab addition showed improvement in ORR (74.4% vs. 51.9%, *P* = 0.00006), complete response rate (11.3% vs. 3.1%), and duration of response (10.6 vs. 9.5 months). PD-L1 CPS score was not an inclusion criterion. Data on PFS and OS have not yet been reported. ATTRACTION-4 was a phase 3 trial centered in Asia for HER-2-negative patients and evaluated front-line nivolumab or placebo in combination with fluoropyrimidine plus platinum^23^. ORR and PFS were improved, but OS did not differ (median OS of 17.5 months vs. median OS of 17.2 months, *P* = 0.26). The role of subsequent therapy (66% received subsequent therapy) is thought to be the reason for lack of impact on OS. PD-L1 in ATTRACTION-4 was not defined by the CPS method. JAVELIN Gastric 100 was a global phase 3 trial that evaluated the role of maintenance avelumab, an anti-PD-L1 agent, compared with continued chemotherapy after 12 weeks of front-line fluoropyrimidine plus platinum in HER-2-negative patients^24^. Median OS was not different between the two groups (median OS of 10.4 vs. 10.9 months, *P* = 0.1779) in all patients. PD-L1 protein expression in at least 1% of tumor cells was considered positive in this study. When evaluated by PD-L1 of at least 1%, median OS did not differ (median OS of 16.2 vs. 17.7 months*, P* = 0.6352). Post hoc analysis classifying patients based on CPS of at least 1 did show a clinical difference of 3 months (median OS of 14.9 vs. 11.6 months). ORIENT-16 initial results were recently reported. ORIENT-16 was a phase 3 trial HER-2-negative Chinese study that evaluated sintilimab, an anti-PD-1 agent, or placebo in combination with fluoropyrimidine plus platinum^25^. PD-L1-positive was not an inclusion criterion. In those with CPS of at least 5, an improvement in OS was seen (18.4 vs. 12.9 months*, P* = 0.0023). An improvement was also seen in all patients (median OS of 15.2 vs. 12.3 months, *P* = 0.0090). OS benefits were seen in all CPS cutoffs (CPS of at least 1, 5, and 10).

CHECKMATE 649 and KEYNOTE-811 led to US Food and Drug Administration approvals for these respective combinations and indications^20,22^. Currently, for HER-2-positive advanced GAC, pembrolizumab plus trastuzumab with fluoropyrimidine and platinum can be a new treatment approach regardless of PD-L1 status. For those with HER-2-negative GACs, nivolumab plus fluoropyrimidine and platinum represents the standard approach for those with PD-L1 CPS >5 disease. JAVELIN Gastric 100 and ATTRACTION-4 revealed the importance of establishing the best method for defining PD-L1 status and the importance of being consistent amongst studies^23,24^. ATTRACTION-4 revealed the importance in the careful selection of the primary outcome objective that will determine the true benefit of the therapy being studied^23^. ORIENT-16 also showed improvement in GAC patients with CPS of at least 5 and all patients^25^. We await the full results and publications of KEYNOTE-811 and ORIENT-16 along with other studies as now we have numerous anti-PD-1 antibodies^22,25^. Of note, Yoon *et al.* recently published a systematic review and meta-analysis of 17 phase 3 randomized trials of immune checkpoint therapy in patients with advanced gastroesophageal cancers (n = 11,166)^26^. This meta-analysis further strengthens that PD-L1 expression and how this relates to response still need to be defined, as differences were seen between histologies.

The abundance of anti-HER-2 agents under development in many tumor types represents an exciting time in oncology. After anti-HER-2 study failures in GAC with lapatinib, pertuzumab, and trastuzumab emtansine, advanced HER-2-positive GAC gained the approval of trastuzumab deruxtecan in January 2021^27–29^. Trastuzumab deruxtecan, an antibody-drug conjugate (ADC) with trastuzumab plus a topoisomerase inhibitor, was approved based on results of the DESTINY-Gastric01 randomized phase 2 trial conducted in the third-line setting^30^. This Asian trial enrolled ERBB2 (HER-2) protein-positive patients who had progressed on two or more prior lines of therapy. HER-2-positive patients who had received at least two prior lines of treatment were given trastuzumab deruxtecan or physician’s choice of chemotherapy. ORR was improved (ORR of 51% vs. 14%), and median OS was improved (median of 12.5 vs. 8.4 months, p = 0.01). Updates on DESTINY-Gastric02, a phase 2 single-arm trastuzumab deruxtecan study, were recently presented^31^. DESTINY-Gastric02 reported on trastuzumab deruxtecan in refractory Western GAC patients (n = 79)^32^. Median OS was 12.1 months, ORR was 41.8%, median duration of response was 8.1 months, and median PFS was 5.6 months. These trials show promise of this agent in multiple patient groups. Phase 3 evaluation via the DESTINY-Gastric04 trial comparing trastuzumab deruxtecan to ramucirumab plus paclitaxel is underway^32^. We sound a note of caution when using trastuzumab-deruxtecan, as this drug has been associated with interstitial lung disease. Other promising combinations for HER-2-directed therapies in GAC show preliminary results. The ZWI-ZW25-201, a phase 2 trial, is evaluating ZW25, a bispecific HER-2 monoclonal antibody, in combination with standard upfront fluoropyrimidine plus platinum^33^. Twenty-eight evaluable patients show an ORR of 75%, and median PFS was 12 months. MAHOGANY is a phase 2/3 trial evaluating margetuximab, an anti-HER-2 monoclonal antibody, in combination with retifanlimab, an anti-PD-L1 agent^34^. Outcomes on 41 patients of the phase 2a cohort A arm of MAHOGANY treated in first line with this chemotherapy-free regimen of margetuximab and retifanlimab showed 78% tumor shrinkage on first scan, ORR of 73% with median duration of response of 10.3 months, disease control rate of 73%, and a median PFS of 6.4 months. There are many other molecules under way for HER-2-directed therapy in early GAC trial exploration, including tyrosine kinase inhibitors (tucatinib, afatinib, poziotinib, pyrotinib), antibodies (margetuximab, ZW25, KN026, KN046, PRS-343, BDC-1001, and MM-111), ADCs (RC48, ZW49, ARX788, SYD985, A1666, XMT-1522, GQ1001, MRG002, PF-06804103, MEDI4276, and ADCT-502), vaccinations (IMU-131 and TAEK-VAC-HERby), and T cell–directed therapy (TAC-01-HER-2, BPX-603, and CCT303-406)^35^. We anticipate more approvals in HER-2-directed therapy for GAC and this subset of patients will soon have a different treatment algorithm.

Claudin 18.2 (CLDN18.2) is an additional target under way in GAC investigation^36,37^. Zolbetuximab, a CLDN18.2 monoclonal antibody, has recently had outcomes reported via monotherapy (MONO trial) and in combination with triplet chemotherapy of epirubicin plus oxaliplatin + fluoropyrimidine compared with chemotherapy alone (FAST trial)^36^. The MONO trial, a phase 2 trial, evaluated zolbetuximab monotherapy in patients with CLDN18.2 expression. Patients had to have at least 50% CLDN18.2 expression of tumor cells. ORR was 9%, and 14% had stable disease^36^. Those with CLDN18.2 expression of at least 70% showed an ORR of 14%, and 17% had stable disease. The FAST trial, a phase 2 trial, showed improvement in PFS and OS with the addition of zolbetuximab (median PFS was 7.5 vs. 5.3 months, *P* <0.0005; median OS of 13.0 vs. 8.3 months, *P* < 0.0005) in all patients (patients were required to have at least 40% CLDN18.2 expression)^37^. Patients with at least 70% of CLDN18.2 showed a median OS of 16.5 months in the zolbetuximab arm. Zolbetuximab is currently being studied in phase 3 trials: the SPOTLIGHT^38^ and GLOW^39^ trials. Additional targeted agents at CLDN18.2, including ADCs, monoclonal antibodies, and through T-cell therapy, are being investigated^39–46^. We look forward to continued study with this unique target for GAC.

Fibroblast growth factor receptor (FGFR) has emerged as an additional relevant target for GAC. The FIGHT trial, a phase 2 trial, evaluated bemarituzumab, an anti-FGFR2b agent, in combination with fluoropyrimidine plus platinum compared with chemotherapy alone^47^. The combination showed improvement in OS (median OS of 19.2 months vs. median OS of 13.5 months). Of interest for those with at least 10% FGFR2b^+^, median OS was 25.4 months vs. 11.1 months in the placebo arm. The combination appears well tolerated, and the addition of ocular toxicity monitoring is likely needed. This combination is undergoing phase 3 exploration via the FORTITUDE-101 study^48^. Bemarituzumab is additionally being studied in other combinations (FORTITUDE-102 and FORTITUDE-103)^49,50^. Other FGFR agents such as multikinase inhibitors (anlotinib and dovitinib) and pan-FGFR inhibitors (HMPL-452, pemigatinib, erdafitinib, and futibatinib) are undergoing investigation^51^. Different toxicity profiles will emerge with further study.

## Conclusion

Molecularly, we are beginning to determine distinctions in targets and in the predictive and prognostic role of these molecular aberrations. We see localized therapy treatment in the coming years to be separated into MSI-H/dMMR and MSI-stable/low/proficient MMR disease. Additionally, answers defining the role of immune checkpoint therapy will emerge along with more insight into immune checkpoint therapy in combination with other mechanisms (e.g., anti-angiogenic agents)^52,53^. We hope that a universal approach to perioperative therapy with emphasis placed on neoadjuvant therapy will be defined, although we recognize this is a far reach. With results of ongoing large phase 3 studies, the best approach, we hope, will emerge^54,55^. For advanced GAC, we are finally at a time when we are beginning to understand this heterogenous disease and seeing that different treatment approaches are needed dependent on the molecular basis of the patient’s disease. We know the coming years will bring newer agents, and we look forward to the outcomes of these current studies.
